# Genetic and Expression Analysis of COPI Genes and Alzheimer’s Disease Susceptibility

**DOI:** 10.3389/fgene.2019.00866

**Published:** 2019-09-19

**Authors:** Yu Yang, Xu Wang, Weina Ju, Li Sun, Haining Zhang

**Affiliations:** Department of Neurology and Neuroscience Center, First Hospital of Jilin University, Changchun, China

**Keywords:** Alzheimer’s disease, COPI, genome-wide association studies, expression quantitative trait loci, gene-based test

## Abstract

Alzheimer’s disease (AD) is the most common neurodegenerative disease in the elderly and the leading cause of dementia in humans. Evidence shows that cellular trafficking and recycling machineries are associated with AD risk. A recent study found that the coat protein complex I (COPI)–dependent trafficking *in vivo* could significantly reduce amyloid plaques in the cortex and hippocampus of neurological in the AD mouse models and identified 12 single-nucleotide polymorphisms in COPI genes to be significantly associated with increased AD risk using 6,795 samples. Here, we used a large-scale GWAS dataset to investigate the potential association between the COPI genes and AD susceptibility by both SNP and gene-based tests. The results showed that only rs9898218 was associated with AD risk with *P* = 0.017. We further conducted an expression quantitative trait loci (eQTLs) analysis and found that rs9898218 G allele was associated with increased *COPZ2* expression in cerebellar cortex with *P* = 0.0184. Importantly, the eQTLs analysis in whole blood further indicated that 11 of these 12 genetic variants could significantly regulate the expression of COPI genes. Hence, these findings may contribute to understand the association between COPI genes and AD susceptibility.

## Introduction

Alzheimer’s disease (AD) is the most common neurodegenerative disease in the elderly and the leading cause of dementia in humans (Jiang et al., 2017; Liu et al., 2017b). It is suggested that genetic risk factors could cause AD (Van Cauwenberghe et al., 2016). In recent years, kinds of methods have been used to detect the underlying AD genetic factors. For example, candidate gene studies have identified mutations in *APP*, *PSEN1*, and *PSEN2* to be associated with autosomal dominant AD (Van Cauwenberghe et al., 2016). *APOE* has been reported to be associated with both early- and late-onset AD (Van Cauwenberghe et al., 2016). Large-scale genome-wide association studies (GWASs) have identified several novel genetic risk loci in European population, and candidate gene studies have replicated these findings in other populations (Liu et al., 2012; Liu et al., 2013b; Liu et al., 2013c; Liu et al., 2013d; Liu et al., 2014a; Liu et al., 2014b; Liu et al., 2014c; Chen et al., 2015; Li et al., 2015; Shen et al., 2015; Zhang et al., 2015; Chang et al., 2016; Li et al., 2016; Liu and Jiang, 2016; Liu et al., 2016; Ma et al., 2016; Tan et al., 2016; Zhang et al., 2016b). Whole-genome sequencing has highlighted the role of *TREM2* in AD (Jiang et al., 2016; Zhang et al., 2016a; Ulland and Colonna, 2018). However, these AD susceptibility loci could only explain 28.57% AD genetic risk (Cuyvers and Sleegers, 2016). Large proportion of AD heritability remains unclear.

It is reported that the cellular trafficking and recycling machineries are associated with AD risk (Bettayeb et al., 2016). Bettayeb et al. (2016) found that the coat protein complex I (COPI)–dependent trafficking *in vivo* could significantly reduce amyloid plaques in the cortex and hippocampus of neurological in the AD mouse models. Bettayeb et al. (2016) further analyzed the human genetic association study data and identified 12 single-nucleotide polymorphisms (SNPs) in COPI genes, which are significantly associated with increased AD risk including *COPA* (rs7531886 and rs12033011), *COPB1* (rs72868007), *COPD/IFT46* (rs73022058 and rs3132828), *COPD/PHLDB1* (rs498872), *COPZ1* (rs34280607 and rs61614746), *COPZ2* (rs757352, rs9898218, rs7216504), and *COPZ2/NFE2L1* (rs11650615).

In their study, Bettayeb et al. Bettayeb et al. (2016) selected six independent study cohorts including two family-based studies and four case-control association studies and further performed a meta-analysis using a total of 6,795 samples (4,018 AD cases and 2,777 controls). Until now, large-scale GWASs have been performed (Harold et al., 2009; Lambert et al., 2009; Holliday et al., 2012; Lambert et al., 2013; Jansen et al., 2019). Hence, we used a large-scale AD GWAS dataset to investigate the association of these 12 genetic variants and the COPI genes and AD risk by a single SNP test and a gene-based test (Lambert et al., 2013). Meanwhile, considering the unknown function of the significant SNP, we conducted an expression quantitative trait loci (eQTLs) analysis.

## Materials and Methods

### AD GWAS Dataset

We selected the AD GWAS dataset from the International Genomics of Alzheimer’s Project (IGAP) (Lambert et al., 2013). International Genomics of Alzheimer’s Project is a large two-stage study based on GWAS on individuals of European ancestry. In stage 1, IGAP used genotyped and imputed data on 7,055,881 SNPs for meta-analysis of four previously published GWAS datasets consisting of 17,008 Alzheimer’s disease cases and 37,154 controls (European Alzheimer’s disease Initiative, Alzheimer Disease Genetics Consortium, Cohorts for Heart and Aging Research in Genomic Epidemiology consortium, Genetic and Environmental Risk in AD consortium) (Lambert et al., 2013). All patients with AD satisfied the National Institute of Neurological and Communicative Disorders and Stroke and the Alzheimer’s Disease and Related Disorders Association criteria or the *Diagnostic and Statistical Manual of Mental Disorders, Fourth Edition* guidelines (Lambert et al., 2013). Previous studies have provided more detailed information about IGAP dataset (Jiang et al., 2017; Liu et al., 2017a; Liu et al., 2018d).

### SNP-Based Test

Here, we investigated the association between these 12 variants and AD susceptibility using the summary association results from the above study (Lambert et al., 2013). If one of these 12 variants is not available in the AD GWAS dataset, we used HaploReg (version 4) to identify the proxy SNPs based on the linkage disequilibrium (LD) information in 1000 Genomes Project (Ward and Kellis, 2012).

### Gene-Based Test

We performed a gene-based test of this large-scale AD GWAS dataset using a common method PLINK (SET SCREEN TEST) (Moskvina et al., 2011). PLINK is a meta-analysis using all the SNPs in the corresponding genes (Moskvina et al., 2011). The method uses an approximate Fisher’s test to combine *P* values across all the SNPs in genes and adjusts for LD (Moskvina et al., 2011). Meanwhile, we performed a gene-based test of this large-scale AD GWAS dataset using VEGAS (Liu et al., 2010). VEGAS software incorporates information from all SNPs within a gene and adjusts the gene sizes, SNP density, and the LD between SNPs (Liu et al., 2010). VEGAS assigns SNPs to 17,787 autosomal genes according to the positions of SNPs and genes (± 50 kb from the 5′ and 3′ UTR) (Liu et al., 2010). Previous studies have provided more detailed information about the PLINK and VEGAS methods (Liu et al., 2013a; Liu et al., 2017d; Li et al., 2018; Lang et al., 2019).

### eQTLs Analysis

We selected the eQTLs dataset from the Brain eQTL Almanac (Braineac), which is a web-based resource to access the UK Brain Expression Consortium dataset (Ramasamy et al., 2014). This resource included 134 neuropathologically normal individuals of European descent in 10 brain tissues (Ramasamy et al., 2014). For each sample, we got his/her COPI gene expression data and the genotype data for 12 SNPs (Ramasamy et al., 2014). We then evaluated their association with nearby gene expression using a linear regression analysis under an additive model. In addition to normal human brain tissues, we further evaluated whether these genetic variants could regulate the expression of nearby genes in neurodegenerative disease tissues. We selected two eQTLs datasets from 197 AD cerebellar samples and 202 AD temporal cortex samples (Zou et al., 2012). The significance level is *P* < 0.05. Recent studies have provided more detailed information about the eQTLs using Braineac (Hu et al., 2017; Liu et al., 2017a; Liu et al., 2017c; Liu et al., 2018a; Liu et al., 2018b; Liu et al., 2018c; Zhang et al., 2018). In addition, we conducted an eQTLs analysis in whole blood using the large-scale dataset from the eQTLGen Consortium (Võsa et al., 2018). The consortium incorporates 37 datasets, with a total of 31,684 individuals (Võsa et al., 2018).

## Results

### SNP-Based Test

Using SNP-based test, we found that 10 of the 12 SNPs were included in this GWAS dataset except rs7531886 and rs34280607 variants. We further applied HaploReg (version 4) to identify their proxy SNPs based on the LD information from the 1000 Genomes Project (EUR) (Ward and Kellis, 2012). We selected two best tagged SNPs including rs2298104 LD with rs7531886 (*r* ^2^ = 0.84 and *D*′ = 0.99), as well as rs34192202 LD with rs34280607 (*r* ^2^ = 0.69 and *D*′ = 0.91). The results indicated that among these 12 SNPs only rs9898218 showed significant association with AD risk with *P* = 0.017, as described in **Table 1**.

**Table 1:** 12 SNPs in COPI genes and Alzheimer’s disease susceptibility.

SNP	Gene	Chr:pos (hg19)	EA	NEA	β	SE	*P* value
rs7531886^a^	COPA	1:160260233	T	C	−0.025	0.016	0.113
rs12033011	COPA	1:160296055	A	G	0.005	0.019	0.806
rs72868007	COPB1	11:14479553	A	C	0.040	0.044	0.364
rs73022058	COPD/IFT46	11:118423672	T	C	−0.025	0.024	0.302
rs3132828	COPD/IFT46	11:118431003	A	G	0.029	0.021	0.164
rs498872	COPD/PHLDB1	11:118477367	A	G	0.011	0.017	0.498
rs34280607^b^	COPZ1	12:54684768	A	G	0.045	0.044	0.305
rs61614746	COPZ1	12:54740214	A	G	−0.019	0.026	0.475
rs757352	COPZ2	17:46097153	A	G	0.017	0.020	0.379
rs9898218	COPZ2	17:46106634	T	G	0.040	0.017	0.017
rs7216504	COPZ2	17:46117341	A	G	0.016	0.019	0.421
rs11650615	COPZ2/NFE2L1	17:46123698	G	C	0.019	0.017	0.289

^a^rs2298104. ^b^rs34192202. EA, effect allele; NEA, noneffect allele; β, overall estimated effect size for the effect allele; SE, overall standard error for effect size estimate; Chr, chromosome; pos, position.

### Gene-Based Test

Using the gene-based test, our results further showed no significant association between these COPI genes and AD susceptibility (significance level 0.05), including *COPA* (*P* = 0.3186 and 0.342), *COPB1* (*P* = 0.6095 and 0.212), *COPD/PHLDB1* (*P* = 0.2942 and 0.325), *COPZ1* (*P* = 0.3803 and 0.454), and *COPZ2* (*P* = 0.08175 and 0.177) using PLINK and VEGAS, respectively.

### eQTLs Analysis

As provided in **Table 1**, rs9898218 is the only SNP associated with AD risk with *P* = 0.017. We first evaluated the association between rs9898218 and *COPZ2* expression using the Braineac dataset. The results showed that rs9898218 G allele was associated with increased *COPZ2* expression in cerebellar cortex (*P* = 1.84E−02), but not in other nine brain tissues (**Table 2**).

**Table 2:** Association between rs9898218 and *COPZ2* expression in Braineac dataset.

SNP	EA	NEA	β	SE	*P* value	Tissue
rs9898218	G	T	0.069	0.029	**1.84E−02**	Cerebellar cortex
rs9898218	G	T	−0.012	0.031	6.94E−01	Frontal cortex
rs9898218	G	T	0.029	0.027	2.79E−01	Hippocampus
rs9898218	G	T	0.003	0.038	9.41E−01	Medulla
rs9898218	G	T	0.028	0.032	3.81E−01	Occipital cortex
rs9898218	G	T	−0.053	0.033	1.14E−01	Putamen
rs9898218	G	T	−0.056	0.032	8.45E−02	Substantia nigra
rs9898218	G	T	0.007	0.033	8.27E−01	Temporal cortex
rs9898218	G	T	0.025	0.031	4.30E−01	Thalamus
rs9898218	G	T	0.024	0.026	3.57E−01	Intralobular white matter

EA, effect allele; NEA, noneffect allele; β, overall estimated effect size for the effect allele; SE, overall standard error for effect size estimate. The significance level is P < 0.05.

In cerebellar cortex, rs2298104 and rs7531886 were associated with the expression of *COPA*, and the rs11650615 was associated with the expression of *COPZ2*. In occipital cortex, both rs12033011 and rs7531886 were associated with the expression of *COPA*. In putamen, rs12033011, rs2298104, and rs7531886 were associated with the expression of *COPA* (**Table 3**).

**Table 3:** P values for the association between other variants excluding rs9898218 and COPI gene expression in the Braineac dataset.

SNP	Gene	Probe ID	CRBL	FCTX	HIPP	MEDU	OCTX	PUTM	SNIG	TCTX	THAL	WHMT
rs12033011	COPA	t2440143	0.23	0.055	0.37	0.096	0.017	0.03	0.23	0.66	0.14	0.43
rs2298104	COPA	t2440143	0.021	0.24	0.31	0.26	0.065	0.006	0.81	0.68	0.4	0.51
rs7531886	COPA	t2440143	0.021	0.23	0.31	0.17	0.025	0.0036	0.89	0.41	0.28	0.54
rs61614746	COPZ1,MIR148B	t3416522	0.44	0.069	0.74	0.69	0.17	0.56	0.41	0.37	0.51	0.71
rs11650615	COPZ2,MIR152	t3761054	0.005	0.93	0.31	0.65	0.47	0.18	0.18	0.93	0.29	0.26
rs757352	COPZ2,MIR152	t3761054	0.1	0.82	0.43	0.44	0.45	0.28	0.32	0.33	0.45	0.36
rs7216504	COPZ2,MIR152	t3761054	0.053	0.8	0.71	0.4	0.51	0.72	0.23	0.64	0.56	0.67

CRBL, cerebellar cortex; FCTX, frontal cortex; HIPP, hippocampus; MEDU, medulla; OCTX, occipital cortex; PUTM, putamen; SNIG, substantia nigra; TCTX, temporal cortex; THAL, thalamus; WHMT, intralobular white matter.

In two AD eQTLs datasets, 3 (rs11650615, rs9898218, and rs498872) of these 12 genetic variants are available. However, none of these three genetic variants was associated with the COPI gene expression in AD cerebellar and temporal cortex tissues (**Table 4**).

**Table 4:** Association between COPI genetic variants and gene expression in AD brain tissues.

SNP	EA	β	*P* value	PROBE	Gene	Tissue
rs11650615	G	−0.049	0.168	ILMN_1667361	COPZ2	AD cerebellar
rs9898218	T	0.009	0.787	ILMN_1667361	COPZ2	AD cerebellar
rs11650615	G	−0.017	0.701	ILMN_1667361	COPZ2	AD temporal cortex
rs9898218	T	−0.008	0.850	ILMN_1667361	COPZ2	AD temporal cortex
rs9898218	T	−0.005	0.787	ILMN_1739450	NFE2L1	AD cerebellar
rs11650615	G	−0.004	0.851	ILMN_1739450	NFE2L1	AD cerebellar
rs11650615	G	−0.019	0.389	ILMN_1739450	NFE2L1	AD temporal cortex
rs9898218	T	−0.014	0.510	ILMN_1739450	NFE2L1	AD temporal cortex
rs498872	A	0.033	0.264	ILMN_1666819	PHLDB1	AD cerebellar
rs498872	A	−0.068	0.068	ILMN_1666819	PHLDB1	AD temporal cortex

EA, effect allele; NEA, noneffect allele; β, overall estimated effect size for the effect allele.

In whole blood, the eQTLs analysis showed that 11 of these 12 genetic variants could significantly regulate the expression of COPI genes including *COPA*, *COPB1*, *COPZ1*, *COPZ2*, *IFT46*, *NFE2L1*, and *PHLDB1* (**Table 5**).

**Table 5:** Association between COPI genetic variants and gene expression in whole blood.

SNP	Study	Tissue	Gene	Ensemble ID	*P* value	Sample
rs7531886	eQTLGen	Whole blood	COPA	ENSG00000122218	2.18E−109	30424
rs12033011	eQTLGen	Whole blood	COPA	ENSG00000122218	4.07E−63	31675
rs72868007	eQTLGen	Whole blood	COPB1	ENSG00000129083	1.03E−34	29143
rs61614746	eQTLGen	Whole blood	COPZ1	ENSG00000111481	2.33E−34	31568
rs757352	eQTLGen	Whole blood	COPZ2	ENSG00000005243	2.42E−04	31680
rs9898218	eQTLGen	Whole blood	COPZ2	ENSG00000005243	1.47E−12	31684
rs7216504	eQTLGen	Whole blood	COPZ2	ENSG00000005243	1.06E−14	27737
rs11650615	eQTLGen	Whole blood	COPZ2	ENSG00000005243	6.53E−36	31562
rs73022058	eQTLGen	Whole blood	IFT46	ENSG00000118096	1.60E−77	28802
rs3132828	eQTLGen	Whole blood	IFT46	ENSG00000118096	8.78E−07	26557
rs757352	eQTLGen	Whole blood	NFE2L1	ENSG00000082641	8.12E−55	14259
rs7216504	eQTLGen	Whole blood	NFE2L1	ENSG00000082641	1.70E−07	10431
rs11650615	eQTLGen	Whole blood	NFE2L1	ENSG00000082641	2.83E−12	14256
rs73022058	eQTLGen	Whole blood	PHLDB1	ENSG00000019144	5.74E−04	28802
rs498872	eQTLGen	Whole blood	PHLDB1	ENSG00000019144	1.12E−04	31300

## Discussion

In recent years, COPI genes have been reported to be potentially involved in AD (Liu et al., 2015). For example, a cluster analysis of microarray data indicated the association between *COPA* and AD (Guttula et al., 2012). Dynamic regulatory network reconstruction analysis showed gradually depressed activity of *COPA* (Kong et al., 2014). Bettayeb et al. (2016) highlighted 12 SNPs including rs7531886, rs12033011, rs72868007, rs73022058, rs3132828, rs498872, rs34280607, rs61614746, rs757352, rs9898218, rs7216504, and rs11650615 in COPI genes *COPA*, *COPB1*, *COPD/IFT46*, *COPD/PHLDB1*, *COPZ1*, *COPZ2*, and *COPZ2/NFE2L1* to be significantly associated with increased AD risk.

With the wide application of GWAS method in AD, it is possible and rapid to validate a finding using large-scale AD GWAS dataset. Here, we selected a large-scale AD GWAS dataset and performed both SNP and gene-based tests. We think that this large-scale dataset may be more powerful than the original dataset (Bettayeb et al., 2016). Using SNP-based test, the results showed that rs9898218 T allele could increase AD risk with β = 0.040 and *P* = 0.017. Two gene-based test methods indicated no significant association between these COPI genes and AD susceptibility. Interestingly, eQTLs analysis further showed that rs9898218 T allele could reduce *COPZ2* expression in cerebellar cortex with β = −0.069 and *P* = 1.84E−02, but not in other nine brain tissues. Meanwhile, we identified other four genetic variants (rs12033011, rs2298104, rs7531886, and rs11650615) regulating the COPI gene expression in other human brain tissues. Importantly, the eQTLs analysis in whole blood further indicated that 11 of these 12 genetic variants could significantly regulate the expression of COPI genes.

*COPZ2* encodes a member of the adaptor complexes small subunit family (Shtutman et al., 2011). Evidence showed down-regulated *COPZ2* expression in most tumor cell lines and in individuals with kinds of cancer types (Shtutman et al., 2011). Interestingly, recent studies have highlighted the role of *COPZ2* in AD (Ciryam et al., 2016; Wan Nasri et al., 2018). Wan Nasri et al. (2018) evaluated the effect of 6 months of tocotrienol rich fraction supplementation on gene expression in the hippocampus of wild-type group (n  =  4) and APPswe/PS1dE9 double transgenic AD mice (n   =  4). They found that Copz2 was significantly down-regulated in AD group compared with the WT group (*P* = 6.44E−05 and fold change = −4.5788) (Wan Nasri et al., 2018). Ciryam et al. (2016) conducted a meta-analysis of gene expression data from about 1,600 human central nervous system tissues to investigate the transcriptional changes upon aging and as a result of AD. They found that *COPZ2* was up-regulated in AD (*P* = 3.90E−05 and fold change = 1.13).

In summary, these findings may provide important information about the association between COPI genes and AD susceptibility. Meanwhile, future studies are required to replicate these findings using large-scale GWAS and eQTLs datasets.

## Data Availability

Publicly available datasets were analyzed in this study. These data can be found here: http://web.pasteur-lille.fr/en/recherche/u744/igap/igap_download.php.

## Author Contributions

LS and HZ proposed the project. YY collected and analyzed the data. All authors wrote the manuscript and approved the final version of the manuscript.

## Conflict of Interest Statement

The authors declare that the research was conducted in the absence of any commercial or financial relationships that could be construed as a potential conflict of interest.
